# Heart Failure Therapy Improves Outcomes in Adults With Congenital Heart Disease and Left Ventricular Dysfunction

**DOI:** 10.1016/j.jacadv.2026.102624

**Published:** 2026-03-25

**Authors:** Zeyad M. Kholeif, Mohamed Ellabbad, William R. Miranda, Luke J. Burchill, Avery A. Clark, Svea Alm, Heidi M. Connolly, Alexander C. Egbe

**Affiliations:** Department of Cardiovascular Medicine, Mayo Clinic, Rochester, Minnesota, USA

**Keywords:** cardiac function, clinical outcomes, heart failure therapy

## Abstract

**Background:**

There are limited data about the role of guideline-directed medical therapy (GDMT) in adults with congenital heart disease presenting with heart failure with reduced ejection fraction.

**Objectives:**

This study aimed to assess the effect of GDMT on cardiac function, congestion, and mortality in adults with congenital heart disease presenting with heart failure with reduced ejection fraction. We hypothesized that GDMT uptitration was associated with improved cardiac function, less congestion, and lower mortality.

**Methods:**

GDMT score, left ventricular ejection fraction (LVEF), and N-terminal pro–b-type brain natriuretic peptide (NT-proBNP) levels were assessed at baseline encounter (baseline GDMT score) and 1-year follow-up. Delta (Δ) GDMT was calculated as GDMT at 1 year minus GDMT at baseline. GDMT uptitration was defined as ΔGDMT score > 0.

**Results:**

Of 327 patients (age 46 ± 16 years; LVEF 31 ± 6%), the baseline GDMT score was 2 (1,3), and 135 (41%) had GDMT uptitration. Although patients with GDMT uptitration had similar LVEF and NT-proBNP levels at baseline encounter compared to patients without GDMT uptitration, the GDMT uptitration group had greater temporal improvement in LVEF (relative ΔLVEF 32 ± 12% vs 15 ± 7%; *P* < 0.001), reduction in NT-proBNP levels (relative ΔNT-proBNP −39 ± 15% vs −21 ± 19%; *P* < 0.001), and a lower 10-year incidence of death/transplant (38% vs 59%; *P* = 0.002). Higher ΔGDMT score (adjusted HR: 0.68 [95% CI: 0.54-0.83]; *P* < 0.001) and ΔLVEF (adjusted HR: 0.79 [95% CI: 0.65-0.96], *P* = 0.002) were associated with a lower risk of death/transplant.

**Conclusions:**

GDMT uptitration was associated with improved cardiac function, less congestion, and a lower incidence of death/transplant. Overall GDMT score was low, and only 41% of patients had GDMT uptitration, suggesting suboptimal therapy and need for optimization of heart failure therapy in this population.

Heart failure (HF) is a growing health and economic burden in the United States and accounts for more than 1.2 million hospital admissions and 0.3 million deaths annually.^1^, ^2^, ^3^, ^4^ About 50% of patients with HF have left ventricular (LV) systolic dysfunction, a disease entity commonly known as HF with reduced ejection fraction (HFrEF).^1^, ^2^, ^3^, ^4^ Among patients with HFrEF, there is an association between the severity of LV systolic dysfunction and the risk of morbidity and mortality.^1^, ^2^, ^3^, ^4^ Medical therapy for HF is associated with improvement in LVEF, and patients with improved LVEF have lower risk of HF hospitalization and mortality.^5^, ^6^, ^7^, ^8^, ^9^, ^10^ As a result, the guidelines for management of HF recommended the use of these therapies in patients with HFrEF, hence the term guideline-directed medical therapy (GDMT).^5^, ^6^, ^7^, ^8^, ^9^, ^10^

Adults with congenital heart disease (CHD) were excluded from the landmark clinical trials that form the basis for GDMT recommendations, and hence there are no large-scale clinical trials assessing the clinical benefits of GDMT in this population.^5^^,^^6^^,^^11^^,^^12^ While several observational studies have assessed the efficacy of GDMT in adults with CHD, the clinical benefit of GDMT has not been consistent across studies.^13^, ^14^, ^15^, ^16^, ^17^, ^18^, ^19^, ^20^ Additionally, it is unknown whether GDMT improves LVEF and whether an improvement in LVEF reduces the risk of mortality as observed in the acquired heart disease population. Some reasons for the inconsistent results of prior studies include the enrollment of a heterogeneous population (with different systemic ventricular morphology and EF) and the lack of standardized criteria for assessing the intensity of HF therapy in the different studies.^13^, ^14^, ^15^, ^16^, ^17^, ^18^, ^19^, ^20^ The current study aims to address these limitations by studying the effect of GDMT (using the standardized GDMT score) on well-defined cohort of CHD patients with HFrEF (biventricular physiology, systemic LV, and LVEF ≤40%). The purpose of this study was to assess the effect of GDMT on cardiac function, congestion, and mortality in adults with CHD and HFrEF. We hypothesized that GDMT uptitration (as measured by GDMT score) was associated with improved cardiac function, less congestion, and lower mortality.

## Methods

### Study population

The Mayo Clinic Institutional Review Board approved this study and waived informed consent for patients who provided research authorization. This is a retrospective cohort study of adults (age ≥18 years) with CHD, biventricular physiology, systemic LV, and LVEF ≤40% who had >1 year of follow-up at Mayo Clinic from January 1, 2003, to December 31, 2023. We included only patients with adequate echocardiographic images for the assessment of LVEF at baseline and follow-up assessment at 1 year. The patients were identified through the Mayo Adult Congenital Heart Disease Registry.^21^

We excluded patients with Fontan physiology, congenitally corrected transposition of great arteries, d-transposition of great arteries status post atrial switch operation, and patients with unrepaired/palliated cyanotic CHD. HFrEF was defined as having B or C HF and LVEF ≤40%.^5^^,^^22^ Stage B HF was defined as the presence of structural heart disease such as reduced LVEF without current or prior history of HF symptoms while stage C HF was defined as the presence of structural heart disease such as reduced LVEF with current or prior history of symptoms.^5^^,^^22^

### GDMT score

The GDMT score, also referred to as the HF Collaboratory score, was developed to provide a standardized framework for assessing the intensity of HF therapy and for comparing HF therapy optimization across different studies.^23^, ^24^, ^25^ The HF medications and point system used for calculating GDMT score are shown in Table 1.^23^, ^24^, ^25^ While the original GDMT score included medications such as hydralazine, isosorbide dinitrate, and ivabradine, these medications were excluded from the GDMT score in this study because they were not routinely used for HF management in our clinical practice.^23^, ^24^, ^25^

**Table 1:** GDMT Score

Drug Class	Dose	Points
Beta-blockers		
	None	0
	<50% maximum dose	1
	≥50% maximum dose	2
ACEI/ARB		
	None	0
	<50% maximum dose	1
	≥50% maximum dose	2
ARNI		
	None	0
	Any dose	3
MRA		
	None	0
	Any dose	2
SGLT2i		
	None	0
	Any dose	1
Maximum score		8

ACEI/ARB = angiotensin-converting enzyme inhibitor/angiotensin 2 receptor blocker; ARNI = angiotensin receptor/neprilysin inhibitor; GDMT = guideline-directed medical therapy; MRA = mineralocorticoid receptor antagonist; SGLT2i = sodium glucose co-transporter 2 inhibitor.^23^, ^24^, ^25^

We calculated GDMT score at baseline encounter (baseline GDMT score) and at 1-year follow-up using each patient’s medication list at these clinical encounters. Δ GDMT score calculated as the difference between GDMT score at baseline and 1-year encounters (GDMT at 1-year follow-up minus GDMT at baseline encounter). We defined GDMT uptitration as Δ GDMT score >0. The patients were dichotomized based on this cutoff value as follows: i) GDMT uptitration**^(+)^** group, defined as patients with ΔGDMT score >0, ii) GDMT uptitration**^(−)^** group, defined as patients with ΔGDMT score ≤0.

### Outcomes

The study outcomes were improvement in cardiac function, congestion, and death/transplant. We assessed improvement in cardiac function as the temporal change in LVEF from the baseline echocardiogram to the echocardiogram performed at 1-year follow-up. In addition to LVEF, we also assessed the following secondary indices of cardiac function: LV global longitudinal strain (GLS), LV end-systolic volume index (ESVi), left atrial reservoir strain, and right ventricular free wall strain (RV FWS). Temporal change in cardiac function was calculated as absolute Δ (cardiac function at 1-year follow-up minus cardiac function at baseline encounter), and as relative Δ ([cardiac function at 1-year follow-up minus cardiac function at baseline encounter] ÷ cardiac function at baseline encounter), whereby positive values signify a temporal improvement in cardiac function.

We assessed congestion using N-terminal pro–b-type brain natriuretic peptide (NT-proBNP) levels measured at baseline encounter and at 1-year follow-up. Similar to cardiac function, temporal change in congestion was calculated as absolute Δ NT-proBNP and relative Δ NT-proBNP, respectively. The occurrence of death/transplant was assessed as a time-to-event outcome and ascertain from 1-year follow-up (time zero) until outcome of interest, last clinical encounter, or December 31, 2023.

### Echocardiography

Comprehensive 2-dimensional, Doppler, and speckle-tracking echocardiography was performed according to contemporary guidelines. Offline image analysis was performed in all patients by research sonographers using the standardized protocol for image analysis in the Mayo Adult Congenital Heart Disease Registry Imaging Core Laboratory.^26^, ^27^, ^28^

In brief, images were acquired with Vivid E9 and E95 (General Electric Co) systems, and 3-beat cine-loop clips were obtained from standard left parasternal and apical windows. These images were exported (DICOM) and then analyzed offline using TomTec (TomTec Imaging Systems). The endocardial border was traced automatically by the software, and the automatic tracings were adjusted manually to ensure optimal tracking throughout the cardiac cycle. Segments were excluded objectively if the software was unable to adequately track the myocardium. Patients with ≥2 nonanalyzable segments were considered ineligible for strain analysis.

LVEDV was derived from manually tracing the endocardial borders at end-diastole representing the maximal volume, LVESV was derived from manually tracing the endocardial borders at end-systole representing the minimum volume, and LVEF was assessed using the biplane Simpson's method using the web-based Echocardiography Information Management System developed at Mayo Clinic to facilitate echocardiogram analysis, interpretation, and reporting. The LV volumes were indexed to body surface area.

### Statistical analysis

Data were presented as mean ± SD, median (Q, Q3), and count (%). Between-group comparisons were based on Fisher exact test, unpaired *t*-test, or Wilcoxon rank sum test, as appropriate. Temporal change in cardiac function indices and NT-proBNP levels was assessed using paired *t*-test, absolute Δ was expressed as mean (95% CI), while relative Δ was expressed as mean ± SD. The cumulative incidence of death/transplant was assessed using Kaplan-Meier analysis, and between-group comparison (GDMT uptitration**^(+)^** vs GDMT uptitration**^(−)^**) was based on log-rank test. To assess the relationship between temporal improvement in LVEF and outcomes (death/transplant), we dichotomized the cohort using the median absolute Δ LVEF, and cumulative incidence of death/transplant was compared between the 2 groups using log-rank test.

Cox regression analysis was used to assess the relationship between GDMT score, temporal change in LVEF (absolute Δ LVEF), and outcomes (death/transplant). The variables used in univariable models were selected based on clinical relevance and included GDMT score (baseline and Δ GDMT scores), echocardiographic indices (RV FWS and LVEF), demographic indices, CHD severity, and comorbidities. Patients were dichotomized based on the date of baseline evaluation (early era defined as baseline encounter between January 1, 2003, and December 31, 2012, vs late era defined as baseline encounter between January 1, 2013 and December 31, 2023). We assessed the effect era by modeling early vs late era in the Cox model.

Variables with *P* < 0.1 on univariable analyses were used to create the multivariable model, and the final variable selection was based on stepwise backward selection, with *P* < 0.1 as the criterion for a variable to remain in the model. The occurrence of death/transplant was assessed as a time-to-event outcomes and ascertain from 1-year follow-up (time zero) until outcome of interest, last clinical encounter, or December 31, 2023. Missing data were handled using conditional imputation. The proportional hazards assumption was evaluated using the Schoenfeld residuals test and visual inspection of the log-log survival curves. We assessed collinearity using variance inflation factor, and we defined significant (≥moderate) as variance inflation factor >5.

On subgroup analysis, separate Cox models were created for the following groups: i) patients with cardiac implantable electronic devices at baseline or received cardiac implantable electronic devices during follow-up; ii) patients without a history of cardiac implantable electronic devices implantation; and iii) patients with cardiac catheterization data. All statistical analyses were performed with BlueSky Statistics software (version. 7.10; BlueSky Statistics LLC), and JMP statistical software (version 17.1.0, JMP Statistical Discovery LLC). *P* value < 0.05 was considered to be statistically significant for all analyses.

## Results

### Baseline characteristics

Of 354 patients with HFrEF, 27 (8%) patients were excluded because of inadequate echocardiographic images (11 patients) and lack of follow-up data at 1 year (16 patients). Overall, there were 327 (4%) patients who met the study inclusion criteria, and the underlying CHD diagnoses are shown in Supplemental Table S1. Of 327 patients, 76 (23%) had mild CHD, 215 (66%) had moderate CHD, and 36 (11%) had complex CHD. Of 327 patients, 50 (15%) had cardiac implantable electronic devices implantation at baseline encounter, and another 44 (14%) underwent cardiac implantable electronic devices implantation during follow-up, leading to an overall cardiac implantable electronic devices prevalence of 29% (94/327). Table 2 shows the baseline characteristics of the cohort. The mean age at baseline encounter was 46 ± 16 years, and 155 (47%) presented with stage B HF while 172 (53%) presented with stage C HF. Of 327 patients, 296 (91%) had NT-proBNP data at baseline encounter, while 272 (83%) had NT proBNP data at baseline encounter and at 1 year.

**Table 2:** Baseline Characteristics

	All (N = 327)	GDMT Uptitration^(+)^ (n = 135, 41%)	GDMT Uptitration^(−)^ (n = 192, 59%)	*P* Value
Demographic indices				
Age (y)	46 ± 16	43 ± 17	48 ± 11	0.02
Male	203 (62%)	84 (62%)	119 (62%)	0.96
Body mass index (kg/m^2^)	27.9 ± 7.3	27.1 ± 6.4	28.1 ± 7.8	0.61
CHD severity				
Mild	76 (23%)	30 (22%)	46 (24%)	0.88
Moderate	215 (66%)	89 (66%)	126 (66%)	
Complex	36 (11%)	16 (12%)	20 (10%)	
Surgical/anatomic data				
CIED implantation	50 (15%)	22 (16%)	28 (15%)	0.74
LVOT disease	66 (20%)	22 (16%)	44 (23%)	0.14
Isolated RH lesion	167 (47%)	103 (54%)	64 (47%)	0.26
Comorbidities				
Hypertension	118 (36%)	44 (33%)	74 (39%)	0.27
Coronary artery disease	44 (14%)	19 (14%)	25 (13%)	0.78
Diabetes	39 (12%)	10 (7%)	29 (15%)	0.03
Chronic kidney disease III-V	44 (14%)	9 (7%)	35 (18%)	0.002
Atrial fibrillation	97 (30%)	37 (27%)	60 (31%)	0.45
Atrial flutter/tachycardia	55 (17%)	22 (16%)	33 (17%)	0.83
Obesity	81 (25%)	32 (24%)	49 (26%)	0.62
Laboratory indices				
GFR (ml/min/1.73 m^2^)	81 (55, 81)	83 (64, 99)	78 (51, 96)	0.04
MELD-XI	11.16 (9.44, 14.29)	10.5 (9.4, 12.9)	11.6 (99.4, 16.2)	0.002
NT-proBNP (pg/mL)	768 (282, 2,393)	665 (284, 2,186)	792 (240, 2,683)	0.11
Echocardiographic data				
Systemic indices				
LA reservoir strain (mL/m^2^)	19 ± 11	22 ± 11	17 ± 10	0.08
LV end-diastolic volume (mL/m^2^)	85 ± 31	82 ± 32	88 ± 38	0.72
LV end-systolic volume(mLL/m^2^)	51 ± 29	49 ± 24	51 ± 32	0.12
LV stroke volume index (mL/m^2^)	40 ± 19	41 ± 16	42 ± 15	0.79
LV ejection fraction (%)	31 ± 6	32 ± 6	33 ± 6	0.53
LV longitudinal strain (%)	−12 ± 3	−12 ± 3	−12 ± 3	0.59
≥Mod mitral regurgitation	39 (12%)	14 (10%)	25 (13%)	0.46
≥Mod aortic regurgitation	26 (8%)	12 (9%)	14 (7%)	0.49
Aortic mean gradient (mm Hg)	12 ± 6	13 ± 7	11 ± 6	0.22
Cardiac index (L/min/m^2^)	3.04 ± 1.02	2.91 ± 0.86	3.09 ± 1.32	0.17
Nonsystemic indices				
RA reservoir strain (%)	21 ± 10	23 ± 12	20 ± 7	0.19
RA mean pressure (mm Hg)	10 ± 5	10 ± 6	11 ± 5	0.55
RV systolic pressure (mm Hg)	48 ± 20	47 ± 19	49 ± 22	0.42
RV free wall strain (%)	−16 ± 5	−16 ± 6	−17 ± 5	0.25
≥Mod tricuspid regurgitation	93 (28%)	30 (22%)	63 (33%)	0.03
≥Mod pulmonary regurgitation	34 (10%)	15 (11%)	19 (10%)	0.72
Aortic mean gradient (mm Hg)	21 ± 10	23 ± 11	19 ± 10	0.11
COA mean gradient (mm Hg)	6 (2, 10)	7 (2,10)	5 (3,9)	0.19
Cardiac catheterization data				
RA mean pressure (mm Hg)	13 ± 6	13 ± 6	14 ± 7	0.41
PA mean pressure (mm Hg)	32 ± 12	30 ± 11	33 ± 15	0.18
PA wedge pressure (mm Hg)	16 ± 8	16 ± 9	17 ± 8	0.60
Cardiac index (L/min/m^2^)	2.46 ± 1.22	2.32 ± 0.94	2.53 ± 1.61	0.53
PVR index (wu m^2^)	4.47 ± 2.08	4.82 ± 2.11	4.01 ± 2.23	0.33

Data were presented as mean ± SD, median (Q, Q3), and count (%). Between-group comparisons were based on Fisher exact test, unpaired *t*-test, or Wilcoxon rank sum test, as appropriate. Isolated right heart disease was defined as patients with CHD diagnoses involving only the right heart without concomitant left heart lesions (mitral valve disease, aortic valve disease, or coarctation of aorta). LVOT disease was defined as having any of the following conditions: aortic valve prosthesis, ≥moderate subvalvular, valvular, or supravalvular aortic stenosis (mean gradient >20 mm Hg) or ≥ moderate aortic regurgitation, in the absence of mitral valve disease.

CHD = congenital heart disease; CIED = cardiac implantable electronic device; GFR = glomerular filtration rate; MELD-XI = model for end-stage liver disease excluding international normalized ratio; NT-proBNP = N-terminal pro–b-type brain natriuretic peptide; RA = right atrium; RH = right heart; RV = right ventricle; PA = pulmonary artery; PVR = pulmonary vascular resistance; LA = left atrium; LV = left ventricle; LVOT = left ventricular outflow tract; other abbreviation as in Table 1.

### GDMT score

Table 3 shows the HF medications at baseline encounter, and the most common HF medications were beta-blockers (N = 163, 50%) and angiotensin-converting enzyme inhibitor/angiotensin 2 receptor blocker (N = 149, 46%). The mean and median GDMT score at baseline encounter were 2.39 ± 1.27 and 2 (1, 3), respectively. The mean and median GDMT score at 1-year follow-up were 3.12 ± 1.29 and 3 (2, 4), respectively. The mean Δ GDMT score for the overall cohort was 0.73 (95% CI: 0.62-0.84).

**Table 3:** GDMT at Baseline Encounter

	All (N = 327)	GDMT Uptitration^(+)^ (n = 135, 41%)	GDMT Uptitration^(−)^ (n = 192, 59%)	*P* Value
Medications				
Beta-blockers	163 (50%)	44 (33%)	119 (62%)	<0.001
ACEI/ARB	149 (46%)	44 (33%)	105 (55%)	<0.001
ARNI	42 (13%)	9 (7%)	33 (17%)	0.03
MRA	51 (16%)	7 (5%)	44 (23%)	<0.001
SGLT2i	43 (13%)	13 (10%)	30 (16%)	0.11
GDMT score				
1	96 (29%)	63 (47%)	33 (17%)	<0.001
2	98 (30%)	40 (30%)	58 (30%)	
3	73 (22%)	19 (14%)	54 (28%)	
4	35 (11%)	8 (6%)	27 (14%)	
5	19 (6%)	5 (4%)	14 (7%)	
6	6 (2%)	0	6 (3%)	

Abbreviations as in Table 1.

**Figure 1:**
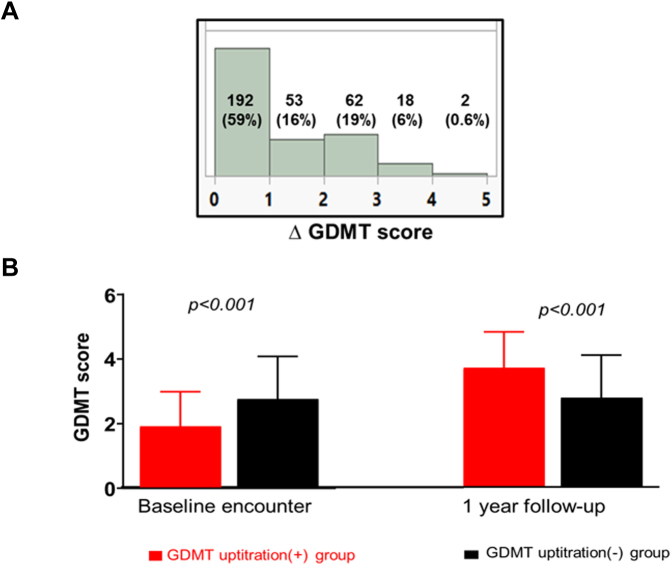
Histogram Showing Use of Guideline-Directed Medical Therapy (A) Histogram showing number of patients who received GDMT uptitration. (B) Box-and-whisker plots comparing GDMT score between patients who received GDMT uptitration (red) vs patients who did not receive GDMT uptitration (black) at baseline encounter and at 1-year follow-up. GDMT = guideline-directed medical therapy.

Figure 1A shows the cohort stratified by Δ GDMT score. Of 327 patients, 135 (41%) had Δ GDMT score >0 (GDMT uptitration**^(+)^** group) while 192 (59%) had Δ ≤ 0 (GDMT uptitration**^(−)^** group). The GDMT uptitration**^(+)^** group had a lower baseline GDMT score compared to the GDMT uptitration**^(−)^** group (mean baseline GDMT score 1.90 ± 1.07 vs 2.73 ± 1.28; *P* < 0.001). However, the GDMT uptitration**^(+)^** group had a mean Δ GDMT of 1.78 (95% CI: 1.65-1.90), leading to a higher GDMT score at 1-year follow-up in the GDMT uptitration**^(+)^** group compared to the GDMT uptitration**^(−)^** group (3.67 ± 1.10 vs 2.73 ± 1.28; *P* < 0.001) (Figure 1B).

At baseline encounter, GDMT uptitration**^(+)^** group vs GDMT uptitration**^(−)^** group had similar LVEF (32 ± 6% vs 33 ± 6; *P* = 0.53), LVGLS (−12 ± 3% vs −12 ± 3; *P* = 0.59), ESVi (49 ± 24% vs 51 ± 32; *P* = 0.12), left atrial reservoir strain (22 ± 11% vs 17 ± 10; *P* = 0.09), RV FWS (−16 ± 6% vs −17 ± 3; *P* = 0.25), and NT-proBNP levels (665 [284, 2,186] vs 792 [240, 2,683] pg/mL; *P* = 0.11) (Table 2).

### Outcomes

#### Temporal change in cardiac function and congestion

Table 4 shows temporal changes in cardiac function and congestion in the overall cohort. There was a temporal improvement in LVEF (absolute Δ 7% [95% CI: 4-10]), and relative Δ 23 ± 11%). Similarly, there was a temporal improvement in other indices of cardiac function (increase LVGLS, left atrial reservoir strain, LV SVi, and decrease in LV ESVi) except RV FWS, and a temporal improvement in congestion (decrease in NT-proBNP level) (Table 4).

**Table 4:** Temporal Change in Echo Indices and Biomarkers

	Baseline (N = 327)	1-Y Follow-Up (N = 327)	Absolute Δ	Relative Δ	*P* Value
LV indices					
LV EDVi (mL/m^2^)	89 ± 32	87 ± 35	−2 (−13 to 7)	−3 ± 9	0.6
LV ESVi (mL/m^2^)	52 ± 24	44 ± 19	−8 (−14 to −2)	−15 ± 9	<0.001
LV SVi (mL/m^2^)	38 ± 12	44 ± 13	6 (1-10)	16 ± 13	0.02
LV EF (%)	31 ± 6	38 ± 8	7 (4-10)	23 ± 11	<0.001
LV GLS (%)	−12 ± 3	−16 ± 4	4 (2-6)	32 ± 14	<0.001
Other echo indices					
LA reservoir strain (%)	19 ± 11	25 ± 10	6 (1-12)	32 ± 14	0.01
RV FWS (%)	−16 ± 5	−19 ± 15	3 (−7 to 1)	17 ± 14	0.09
Biomarkers					
NT-proBNP (pg/mL)	768 (282, 2,393)	529 (219, 1859)	−239 (−411 to 127)	−31 ± 14	<0.001

Δ NT-proBNP was calculated in the subgroup of patients with NT-proBNP measurements at baseline and 1-y follow-up (N = 272, 83%).

EDVi = end-diastolic volume index; EF = ejection fraction; ESVi = end-systolic volume index; FWS = free wall strain; GLS = global longitudinal strain; SVi= stroke volume index; other abbreviations as in Table 2.

**Figure 2:**
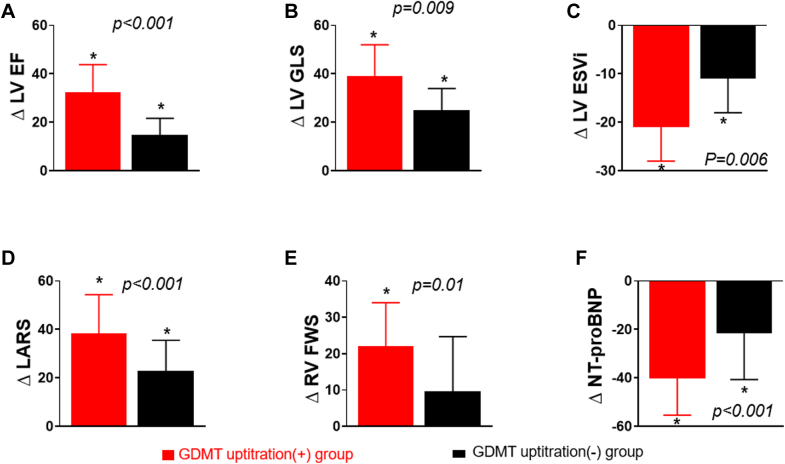
Cardiac Remodeling and Temporal Change in Congestion Box-and-whisker plots comparing cardiac remodeling (A-E) and temporal change in congestion (F) between patients who received GDMT uptitration (red) vs patients who did not receive GDMT uptitration (black) at baseline encounter and at 1-year follow-up. Cardiac remodeling indices were expressed as relative Δ from baseline values. *Signifies statistically significant difference from baseline values. ESVi = end-systolic volume index; FWS = free wall strain; GLS = global longitudinal strain; LARS = left atrial reservoir strain; LV = left ventricle; LVEF = left ventricular ejection fraction; NT-proBNP = N-terminal pro B type brain natriuretic peptide; RV = right ventricle; other abbreviation as in Figure 1.

Compared to the GDMT uptitration**^(−)^** group, patients in the GDMT uptitration**^(+)^** group had greater temporal improvement in LVEF (relative Δ LVEF 32 ± 12% vs 15 ± 7%; *P* < 0.001), LVGLS (relative Δ LVGLS 39 ± 13% vs 25 ± 9%; *P* = 0.009), LV ESVi (relative Δ LV ESVi −21 ± 8% vs −11 ± 7%; *P* = 0.006), left atrial reservoir strain (relative Δ left atrial reservoir strain 38 ± 16% vs 23 ± 13%; *P* < 0.001), RVFWS (relative Δ RVFWS 22 ± 12% vs 9 ± 13%; *P* = 0.01), and NT-proBNP (relative Δ NT-proBNP −39 ± 15% vs −21 ± 19%; *P* < 0.001), (Figure 2).

#### All-cause mortality

Of 327 patients, 133 (41%) died and 17 (5%) underwent heart transplantation during a median follow-up of 5.9 (3.1, 9.6) years. The composite outcome of death/transplant occurred in 143 (44%) patients. Compared to the patients who survived without heart transplant (N = 184, 56%), those who died/underwent heart transplant (N = 143, 44%) had lower GDMT score at baseline encounter (1.98 ± 1.14 vs 2.54 ± 1.21; *P* = 0.03), lower GDMT score at 1-year encounter (2.71 ± 1.33 vs 3.49 ± 1.18; *P* = 0.002), and lower ΔGDMT score (0.56 ± 0.22 vs 0.95 ± 10.31; *P* < 0.00).

The 10-year cumulative incidence of death/transplant was lower in the GDMT uptitration**^(+)^** group compared to others (38% vs 49%; *P* = 0.002). Similarly, 15-year cumulative incidence of death/transplant was lower in the patients with greater improvement in LVEF (absolute Δ LVEF >7%) compared to others (38% vs 60%; *P* = 0.001) (Central illustration).

On multivariable analysis, higher baseline GDMT score (adjusted HR: 0.71 [95% CI: 0.60-0.82] *P* = 0.004, per unit increase), greater GDMT uptitration (ie, higher Δ GDMT score) (adjusted HR: 0.68 [95% CI: 0.54-0.83], *P* < 0.001, per unit increase), and greater temporal improvement in LVEF (ie, higher absolute Δ LVEF) (adjusted HR: 0.79 [95% CI: 0.65-0.96], *P* = 0.008, per 5%-point increase) were associated with lower risk of death/transplant (Table 5 and Central Illustration). Of note, 6 patients underwent cardiac interventions during follow-up (transcatheter pulmonary valve replacement [N = 4], catheter ablation for cavotricuspid isthmus dependent flutter [N = 1], and pulmonary vein isolation for atrial fibrillation [N = 1]). Cardiac intervention was not associated with death/transplant.

**Table 5:** Cox Regression Model Showing Correlates of Death/Transplant

	Univariable		Multivariable	
	HR (95% CI)	*P* Value	HR (95% CI)	*P* Value
GDMT score				
GDMT score at baseline	0.79 (0.68-0.90)	<0.001	0.71 (0.60-0.82)	0.004
Δ GDMT score	0.68 (0.56-0.82)	<0.001	0.68 (0.54-0.83)	<0.001
Echo indices				
LVEF at baseline (per 5%)	0.85 (0.72-0.97)	0.01		
RV FWS at baseline (per 5%)	0.87 (0.75-0.99)	0.02		
Δ LVEF (per 5%)	0.67 (0.56-0.79)	<0.001	0.79 (0.65-0.96)	0.002
Demographic/surgical indices				
Age (per 5 y)	1.09 (1.04-1.15)	<0.001	1.07 (1.02-1.23)	0.008
Male sex	0.82 (0.59-1.48)	0.25		
CHD severity				
Simple	Reference			
Moderate	0.96 (0.67-1.45)	0.92		
Complex	1.31 (0.73-2.30)	0.21		
Comorbidities				
Hypertension	1.35 (1.02-1.64)	0.02		
Coronary artery disease	1.56 (1.02-2.39)	0.04		
Diabetes	1.66 (1.06-2.60)	0.03		
Atrial fibrillation	1.31 (1.03-1.61)	0.01		
Atrial flutter/tachycardia	1.13 (0.93-1.33)	0.18		
Study era				
Baseline encounter in early era (vs late era)	0.96 (0.73-1.18)	0.33		
Cardiac interventions^a^				
CIED at baseline	1.21 (0.84-1.61)	0.39		
CIED during follow-up	0.91 (0.66-1.34)	0.47		
Transcatheter PVR during follow-up	1.08 (0.67-1.43)	0.38		
Catheter ablation during follow-up	0.98 (0.42-1.55)	0.61		

LVEF = left ventricle ejection fraction; RV FEW = right ventricular free wall strain; other abbreviations as in Tables 1 and 2.

a Covariates with *P* < 0.1 on univariable analysis were used to create the multivariable model, and the final covariate selection was based on stepwise backward selection with *P* < 0.1 required for a covariate to remain in the multivariable model. --- signifies covariates with *P* ≥ 0.1. Cardiac intervention was modeled as time-dependent covariates. Patients were dichotomized based on the date of baseline evaluation (early era defined as baseline encounter between January 1, 2003, and December 31, 2012, vs late era defined as baseline encounter between January 1, 2013, and December 31, 2023). We assessed the effect era by modeling early vs late era in the Cox model.

**Central Illustration:**
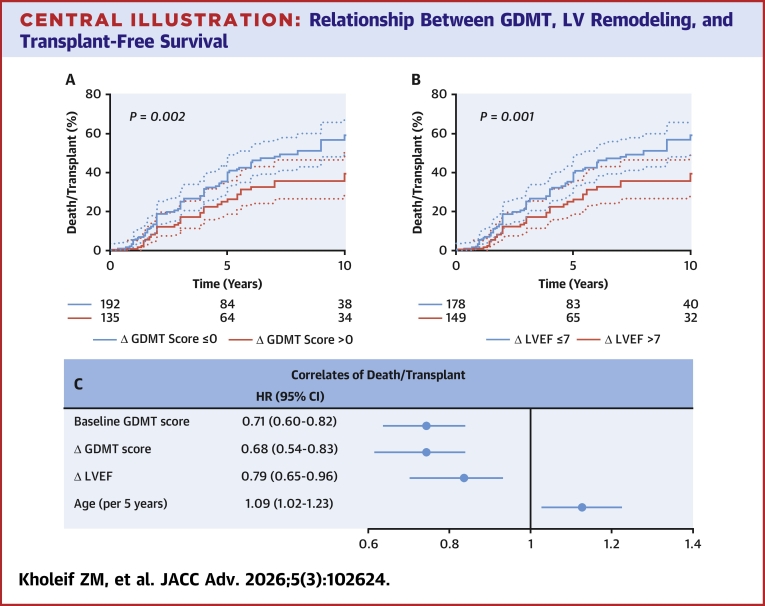
**Relationship Between GDMT, LV Remodeling, and Transplant-Free Survival** Kaplan-Meier curves comparing cumulative incidence of death/transplant between patients who received GDMT uptitration (red) vs patients who did not receive GDMT uptitration (black) (A), and between patients with absolute Δ LVEF >7% (red) vs patients with absolute Δ LVEF ≤7% (black) (B). (C) Forest plot showing correlates of death and transplant. Baseline GDMT score, GDMT uptitration, and temporal increase in LVEF were independently associated with lower risk of death/transplant. See Table 5 for the full Cox regression model. Abbreviations as in Figures 1 and 2.

#### Subgroup analysis

Of the 327 patients, 94 patients had cardiac implantable electronic devices (dual-chamber pacemaker [N = 13], implantable cardioverter-defibrillator [N = 57], biventricular pacemaker/defibrillator [N24]). Among patients without cardiac implantable electronic devices (N = 233), higher Δ GDMT (adjusted HR: 0.68 [95% CI: 0.54-0.83; *P* < 0.001, per unit increase) and higher Δ LVEF (adjusted HR: 0.72 [95% CI: 0.52-0.91; *P* = 0.009, per 5%-point increase in Δ LVEF) were associated with lower risk of death/transplant (Table 6). However, Δ LVEF (adjusted HR: 0.71 [95% CI: 0.46-0.97; *P* = 0.03, per 5%-point increase in Δ LVEF) but not Δ GDMT was associated with death/transplant in patients with cardiac implantable electronic devices (N = 94).

**Table 6:** Cox Regression Model Showing Correlates of Death/Transplant—Subgroup Analysis

	Patients Without CIED (N = 233)		Patients With CIED (N = 94)		Patients With Cath Data (N = 221)	
	HR (95% CI)	*P* Value	HR (95% CI)	*P* Value	HR (95% CI)	*P* Value
GDMT score						
GDMT score at baseline	0.76 (0.63-0.93)	0.007	-	-	-	-
Δ GDMT score	0.70 (0.54-0.89)	0.006	0.76 (0.43-1.04)	0.09	0.70 (0.55-0.98)	0.003
Echo indices						
LVEF at baseline (per 5%)	-	-				
RV FWS at baseline (per 5%)	-	-	0.82 (0.51-0.98)	0.02	-	-
Δ LVEF (per 5%)	0.72 (0.52-0.91)	0.009	0.71 (0.46-0.97)	0.03	0.78 (0.55-1.02)	0.08
Demographic/surgical indices						
Age (per 5 y)	1.10 (1.03-1.17)	0.003	-	-	-	-
Comorbidities						
Hypertension	-	-	-	-	-	-
Coronary artery disease	-	-	-	-	-	-
Diabetes	-	-	-	-	-	-
Atrial fibrillation	-	-	-	-	-	-
Invasive hemodynamic indices						
RA mean pressure (mm Hg)	N/A		N/A		-	-
PA mean pressure (mm Hg)	N/A		N/A		1.03 (1.01-1.05)	0.01
PA wedge pressure (mm Hg)	N/A		N/A		-	-

N/A = not applicable; other abbreviations as in Tables 1, 2, 4 and 5.

Among patients with cardiac catheterization data (N = 221), Δ GDMT (adjusted HR: 0.70 [95% CI: 0.55-0.98; *P* = 0.003, per unit increase) and pulmonary artery mean pressure (adjusted HR: 1.03 [95% CI: 1.01-1.05; *P* = 0.01, per 1 mm Hg) were associated with lower risk of death/transplant (Table 6).

## Discussion

In this study, we assessed the effect of GDMT on cardiac function, congestion, and mortality in adults with CHD and systemic LV presenting with HFrEF. All patients were on GDMT at baseline encounter, but only 41% had GDMT uptitration. Compared to patients without GDMT uptitration, those with GDMT uptitration had greater improvement in cardiac function, less congestion, and lower incidence of death/transplant. Both GDMT uptitration and temporal improvement in LVEF were associated with lower risk of death/transplant.

In patients with HFrEF from acquired heart disease, GDMT is associated with improvement in LVEF and reduction in HF hospitalization and mortality.^5^, ^6^, ^7^, ^8^, ^9^, ^10^ In contrast, the relationship between GDMT and improvement in LVEF and clinical outcomes in adults with CHD is less well defined.^14^^,^^18^, ^19^, ^20^ In a recent study, Neijenhuis et al demonstrated that CHD patients who were already on GDMT had a 3-fold reduction in HF hospitalization following the initiation of sodium glucose co-transporter 2 inhibitor.^14^ In this series, there was no significant improvement in systemic ventricular EF despite significant reduction in HF hospitalization.^14^ Similarly, Maurer et al did not observe any improvement in systemic ventricular EF and functional capacity following initiation of GDMT in CHD patients.^19^ In contrast, Nederend et al and Fusco et al reported improvement in systemic ventricular EF, 6-minute walk distance, and congestion (lower NT-proBNP level) following initiation of GDMT.^18^^,^^20^ These variations in outcome following initiation of GDMT from different studies are related to heterogeneity in the clinical characteristics of the CHD patients enrolled in the studies, and differences in the intensity of GDMT used in these studies.^13^, ^14^, ^15^, ^16^, ^17^, ^18^, ^19^, ^20^ The observed clinical response to GDMT will depend on systemic ventricular morphology and EF, as well as the dose and number of HF medications used in the different studies.^13^, ^14^, ^15^, ^16^, ^17^, ^18^, ^19^, ^20^ The current study overcame these limitations by restricting the inclusion criteria to CHD patients with morphologic LV and HFrEF and by assessing the intensity of medical therapy using the standardized GDMT score. It is noteworthy that while GDMT was associated with improved survival, the mortality in the overall cohort was high (41% of the cohort died in 5 years) compared to mortality rate of less 20% in 5 years in the general adults CHD cohort, highlighting the negative prognostic significance of LV systolic dysfunction. On the other hand, the 5-year mortality rate observed in the current study was significantly lower than 75% mortality at 5 years in HFrEF from acquired heart disease, likely due to older age and more comorbidities in the acquired heart disease population.^13^^,^^29^

Another important finding from the current study was the improvement in RV systolic function especially in the patients who received GDMT uptitration. This may indicate that GDMT was effective in improving RV systolic function or may be due to improvement in RV systolic function following improvement in LVEF (ventricular interaction).

### Clinical implications and future directions

The improvement in cardiac function, congestion, and survival following GDMT uptitration observed in the current study provides empirical evidence for optimization of GDMT in adults with CHD presenting with HFrEF. Although all patients were on GDMT, the median GDMT score was 2, which is lower than the median score of 4 in studies from the acquired heart disease population, and only 41% had GDMT optimization within the first year. Furthermore, there was underutilization the newer HF medications such as the angiotensin receptor/neprilysin inhibitor and the sodium glucose co-transporter 2 inhibitor. Collectively, these findings suggest suboptimal therapy and need for improvement. One of the concerns regarding intensification of HF therapy includes risk of side effects and cost. While the current study did not assess side effects and tolerability of GDMT, we assume that therapy was well tolerated since none of the patients had a lower GDMT score at 1-year follow-up. This is also consistent with previous studies showing that GDMT was well tolerated in adults with CHD.^13^, ^14^, ^15^, ^16^, ^17^, ^18^, ^19^, ^20^ With regard to issue of cost, GDMT has been shown to reduce health care cost and hence beneficial to the patient and the health care system.^30^

### Study Limitations

The strengths of the current study include well-defined inclusion criteria, a relatively large sample size, and a long duration of follow-up. The limitations include retrospective study design, which in turn, has inherent selection and ascertainment bias. Furthermore, we were unable to ascertain compliance with medical therapy or medication intolerance due to the retrospective study design. Additionally, GDMT titration was not randomly assigned, and hence the intensity of therapy may reflect day practice preferences of the primary cardiologist and/or patients’ tolerance to medications. These issues highlight the need for a randomized controlled trial to address these confounders. Pacemaker implantation can have diametrically opposite effects of HF and LVEF, whereby some patients will experience deterioration in LVEF and worsening heart failure because of chronic RV pacing, while other patients may experience improvement in LVEF and heart failure symptoms from physiologic pacing. We controlled these confounders by performing subgroup analysis in patients with vs without cardiac implantable electronic devices.

## Conclusions

GDMT uptitration was associated with improvement in cardiac function, less congestion, and lower incidence of death/transplant in adults with CHD and systemic LV presenting with HFrEF. GDMT uptitration and temporal improvement in LVEF were associated with a lower risk of death/transplant. The overall GDMT score was low, and less than half of the patients had GDMT uptitration.

Perspectives Among adults with CHD presenting with HFrEF, the patients with GDMT uptitration (N = 135, 41%) had greater improvement in cardiac function, less congestion, and lower incidence of death/transplant compared to patients without GDMT (N = 192, 59%). GDMT uptitration and temporal improvement in LVEF were associated with a lower risk of death/transplant. The overall GDMT score was low, relative to historical cohort, and only 41% of patients had GDMT uptitration. Collectively, these data support the use of GDMT for the management of HFrEF in adults with CHD and underscores the need for optimization of HF therapy to improve outcomes.

## Funding support and author disclosures

Dr Egbe is supported by the 10.13039/100000050National Heart, Lung, and Blood Institute (NHLBI) grants (R01 HL158517, R01 HL160761, and R01 HL162830). The MACHD Registry is supported by the Al-Bahar Research grant. The authors have reported that they have no relationships relevant to the contents of this paper to disclose.

## References

[1] Heidenreich P.A., Albert N.M., Allen L.A. (2013). Forecasting the impact of heart failure in the United States: a policy statement from the American Heart Association. Circ Heart Fail.

[2] Virani S.S., Alonso A., Aparicio H.J. (2021). Heart disease and stroke statistics-2021 update: a report from the American Heart Association. Circulation.

[3] Ni H., Xu J. (2015). NCHS Data Brief.

[4] Agarwal M.A., Fonarow G.C., Ziaeian B. (2021). National trends in heart failure hospitalizations and readmissions from 2010 to 2017. JAMA Cardiol.

[5] Heidenreich P.A., Bozkurt B., Aguilar D. (2022). 2022 AHA/ACC/HFSA Guideline for the Management of Heart Failure: a report of the American college of Cardiology/American Heart Association Joint Committee on Clinical Practice Guidelines. J Am Coll Cardiol.

[6] Authors/Task Force M., McDonagh T.A., Metra M. (2022). 2021 ESC Guidelines for the diagnosis and treatment of acute and chronic heart failure: developed by the Task Force for the diagnosis and treatment of acute and chronic heart failure of the European Society of Cardiology (ESC). With the special contribution of the Heart Failure Association (HFA) of the ESC. Eur J Heart Fail.

[7] Cleland J.G.F., Bunting K.V., Flather M.D. (2018). Beta-blockers for heart failure with reduced, mid-range, and preserved ejection fraction: an individual patient-level analysis of double-blind randomized trials. Eur J Heart Fail.

[8] McMurray J.J., Packer M., Desai A.S. (2014). Angiotensin-neprilysin inhibition versus enalapril in heart failure. N Engl J Med.

[9] Pitt B., Zannad F., Remme W.J. (1999). The effect of spironolactone on morbidity and mortality in patients with severe heart failure. Randomized Aldactone Evaluation Study Investigators. N Engl J Med.

[10] McMurray J.J.V., Solomon S.D., Inzucchi S.E. (2019). Dapagliflozin in patients with heart failure and reduced ejection fraction. N Engl J Med.

[11] Stout K.K., Daniels C.J., Aboulhosn J.A. (2019). 2018 AHA/ACC Guideline for the management of adults with congenital heart disease: a report of the American College of Cardiology/American Heart Association task force on clinical practice Guidelines. J Am Coll Cardiol.

[12] Baumgartner H., De Backer J. (2020). The ESC Clinical Practice Guidelines for the management of adult congenital heart disease 2020. Eur Heart J.

[13] Egbe A.C., Miranda W.R., Pellikka P.A., DeSimone C.V., Connolly H.M. (2022). Prevalence and prognostic implications of left ventricular systolic dysfunction in adults with congenital heart disease. J Am Coll Cardiol.

[14] Neijenhuis R.M.L., MacDonald S.T., Zemrak F. (2024). Effect of sodium-glucose cotransporter 2 inhibitors in adults with congenital heart disease. J Am Coll Cardiol.

[15] Woudstra O.I., Kuijpers J.M., Meijboom F.J. (2019). High burden of drug therapy in adult congenital heart disease: polypharmacy as marker of morbidity and mortality. Eur Heart J Cardiovasc Pharmacother.

[16] Ladouceur M., Valdeolmillos E., Karsenty C., Hascoet S., Moceri P., Le Gloan L. (2023). Cardiac drugs in ACHD cardiovascular medicine. J Cardiovasc Dev Dis.

[17] Andi K., Abozied O., Miranda W.R. (2023). Clinical benefits of angiotensin receptor-Neprilysin inhibitor in adults with congenital heart disease. Int J Cardiol.

[18] Nederend M., Kies P., Regeer M.V. (2023). Tolerability and beneficial effects of sacubitril/valsartan on systemic right ventricular failure. Heart.

[19] Maurer S.J., Pujol S.C., Schiele S., Hager A., Ewert P., Tutarel O. (2020). Sacubitril/valsartan for heart failure in adults with complex congenital heart disease. Int J Cardiol.

[20] Fusco F., Scognamiglio G., Merola A. (2023). Safety and efficacy of Sacubitril/Valsartan in patients with a failing systemic right ventricle: a prospective single-center Study. Circ Heart Fail.

[21] Egbe A.C., Miranda W.R., Connolly H.M. (2025). The Mayo Adult Congenital Heart Disease (MACHD) registry and biobank. JACC Adv.

[22] Egbe A.C., Connolly H.M. (2024). Heart failure staging and indications for advanced therapies in adults with congenital heart disease. Heart Fail Clin.

[23] Abraham W.T., Psotka M.A., Fiuzat M. (2020). Standardized definitions for evaluation of heart failure therapies: scientific expert Panel from the heart failure collaboratory and academic research consortium. JACC Heart Fail.

[24] DeFilippis E.M., Fiuzat M. (2021). Putting the “Optimal” in optimal medical therapy. JACC Heart Fail.

[25] Fiuzat M., Hamo C.E., Butler J. (2022). Optimal background pharmacological therapy for heart failure patients in clinical trials: JACC review topic of the week. J Am Coll Cardiol.

[26] Egbe A.C., Miranda W.R., Warnes C.A. (2021). Persistent hypertension and left ventricular hypertrophy after repair of native coarctation of Aorta in adults. Hypertension.

[27] Egbe A.C., Miranda W.R., Jain C.C. (2023). Prognostic performance of right ventricular global longitudinal strain measurements in patients with Ebstein Anomaly. J Am Coll Cardiol.

[28] Egbe A.C., Miranda W.R., Jain C.C., Connolly H.M. (2022). Prognostic implications of progressive systemic ventricular dysfunction in congenitally corrected transposition of great arteries. JACC Cardiovasc Imaging.

[29] Shah K.S., Xu H., Matsouaka R.A. (2017). Heart failure with preserved, borderline, and reduced ejection fraction: 5-year outcomes. J Am Coll Cardiol.

[30] Polsinelli V.B., Sun J.L., Greene S.J. (2024). Hospital heart failure medical therapy Score and associated clinical outcomes and costs. JAMA Cardiol.

