# Supplementary material for: Heart Failure Therapy Improves Outcomes in Adults With Congenital Heart Disease and Left Ventricular Dysfunction
Source: JACC Adv. 2026 Mar 25;5(3):102624. doi: 10.1016/j.jacadv.2026.102624 (PMC13352024; doi:10.1016/j.jacadv.2026.102624)
Supplement: Supplemental Material [file mmc1.docx]

**Supplementary Table S1: CHD Diagnoses**

| **CHD Diagnosis** | **HFrEF**  **(N=327, 4%)** |
| --- | --- |
| Tetralogy of Fallot | 63 (19%) |
| Ebstein | 28 (9%) |
| Pulm valve stenosis | 15 (5%) |
| Pulmonary atresia -IVS | 4 (1.1%) |
| Truncus arteriosus | 3 (0.9%) |
| TGA- arterial switch op | 6 (1.8%) |
| TGA- Rastelli op | 3 (0.9%) |
| Double outlet RV | 6(1.8%) |
| Coarctation of aorta | 44 (14%) |
| Mitral stenosis | 0 |
| Subaortic stenosis | 4 (1.2%) |
| Aortic valve stenosis | 37 (11%) |
| PAPVR | 24 (11%) |
| Atrial septal defect | 33 (10%) |
| Ventricular septal defect | 327 (11%) |
| Atrioventricular canal defect | 13 (4%) |
| Cor triatriatum | 2 (0.6%) |

**Abbreviations**: AS: Aortic stenosis; CHD: Congenital heart disease; HFrEF: Heart Failure with reduced ejection fraction; IVS: Intact ventricular septum; RV: Right ventricle; TGA: Transposition of great arteries; PAPVR: Partial anomalous pulmonary venous return.
